# Urotensin-II: More Than a Mediator for Kidney

**DOI:** 10.1155/2012/249790

**Published:** 2012-10-10

**Authors:** Ayşe Balat, Mithat Büyükçelik

**Affiliations:** ^1^Division of Pediatric Nephrology, Faculty of Medicine, University of Gaziantep, 27310 Gaziantep, Turkey; ^2^Division of Pediatric Nephrology and Rheumatology, Faculty of Medicine, University of Gaziantep, 27310 Gaziantep, Turkey

## Abstract

Human urotensin-II (hU-II) is one of the most potent vasoconstrictors in mammals. Although both hU-II and its receptor, GPR14, are detected in several tissues, kidney is a major source of U-II in humans. Recent studies suggest that U-II may have a possible autocrine/paracrine functions in kidney and may be an important target molecule in studying renal pathophysiology. It has several effects on tubular transport and probably has active role in renal hemodynamics. Although it is an important peptide in renal physiology, certain diseases, such as hypertension and glomerulonephritis, may alter the expression of U-II. As might be expected, oxidative stress, mediators, and inflammation are like a devil's triangle in kidney diseases, mostly they induce each other. Since there is a complex relationship between U-II and oxidative stress, and other mediators, such as transforming growth factor *β* 1 and angiotensin II, U-II is more than a mediator in glomerular diseases. Although it is an ancient peptide, known for 31 years, it looks like that U-II will continue to give new messages as well as raising more questions as research on it increases. In this paper, we mainly discuss the possible role of U-II on renal physiology and its effect on kidney diseases.

## 1. Introduction

Although urotensin-II was firstly identified in a neurohemal organ of fish in 1980s [1, 2], only recently it became a major focus of clinical and experimental researches [3].

Human urotensin-II (hU-II) is a cyclic peptide of 11 amino acids cleaved from a larger prepro-U-II precursor peptide of about 130 amino acids [1, 3, 4]. The gene encoding this peptide is located at 1p36 and contains 5 exons [5]. It is a ligand for the orphan G-protein-coupled receptor, known as GPR14 [3, 6, 7]. 

Although human prepro-U-II mRNA is expressed mainly in the brain and spinal cord [4, 8], both hU-II and its receptor are also detected in other organs and tissues, such as kidney, spleen, smooth muscle, endothelium, small intestine, thymus, prostate, pituitary, and adrenal gland [1, 9–11]. 

Being almost tenfold more potent than endothelin-I (ET), it is the most potent mammalian vasoconstrictor identified to date [3, 12]. It circulates in the plasma of healthy individuals and acts as a circulating vasoactive hormone and as a locally acting paracrine or autocrine factor in cardiovascular regulation [4, 13]. 

Although it mainly has a vasoconstrictor effect, regional differences may be seen in its effects in various vascular beds and blood vessels of some species. For example, it has a vasodilatory effect on the small arteries of rats [14, 15], and on the resistance arteries of humans, through release of endothelium-derived hyperpolarizing factor (EDHF), nitric oxide (NO) [14–16]. 

It has been shown that the potent vasoconstrictor actions of U-II is mediated by Ca^+2^ mobilization through activation of a number of signaling pathways including Ca^+2^ channels, tyrosine kinase, p38 mitogen-activated protein kinase (p38MAPK), and extracellular signal-regulated kinase 1 and 2 (ERK1/2) [17, 18].

Since U-II and its receptor have been demonstrated in mouse, monkey [19], and human kidneys [20, 21], it is acceptable to consider that U-II is synthesized, secreted, and cleared by the kidneys [6, 22–24]. 

Interestingly, Mosenkis et al. [25] showed that hU-II was also present in 2 surgically anephric subjects. Although this finding is inconsistent with the conclusion that the kidneys are the primary source for production of U-II, as the authors stressed, the high density of U-II and its receptor in renal tissues suggest that U-II is metabolically active in the kidney even though it is produced in outside of the kidneys. 

In this paper, we mainly discuss the possible role of U-II on renal physiology and its effect on kidney diseases. 

## 2. Effect of U-II on Renal Hemodynamics 

 Because of its potent vasoconstrictor effect, U-II attracted the interest of researchers in general hemodynamy. In fact, hemodynamic responses to U-II show regional heterogeneity in relation to its receptor localization, even in the differences of functional state of the endothelium [26].

However, in contrast to animal studies, Wilkinson et al. found no vasoactive responses to hU-II in vivo in man [27]. They injected hU-II intra-arterially to healthy male volunteers, and despite the high-circulating hU-II levels, no change was seen in systemic hemodynamics, ECGs of subjects, and hU-II had no effect on hand vein diameter. 

However, another study published in the same year [28] demonstrated that U-II produces potent vasoconstriction in humans in vivo. They showed that U-II induced dose-dependent reduction in forearm blood flow (FBF) of healthy volunteers, and FBF returned to baseline values within 30 min. 

Known data in the literature show that the kidney is a major source of U-II in humans [29], primates, mice [19], and rats [11]. It has been found in the urine of humans [22, 24] and rats [11] at a concentration far exceeding that of plasma. In humans, the renal clearance of U-II has found to be greater than that of creatinine, suggesting that urinary U-II is derived primarily from the kidney [22]. An animal study also revealed that there was an arteriovenous concentration gradient for U-II across the renal circulation in anaesthetised sheep [30]. As well as U-II, its receptor has also been localized to the mammalian kidneys, such as in human [22], monkey, mouse [19], and rat kidneys [11]. It has been shown that the medulla, especially tubular component of the kidney, is the principal site of U-II receptor expression in the rat kidney [11, 31]. 

**Figure 1:**
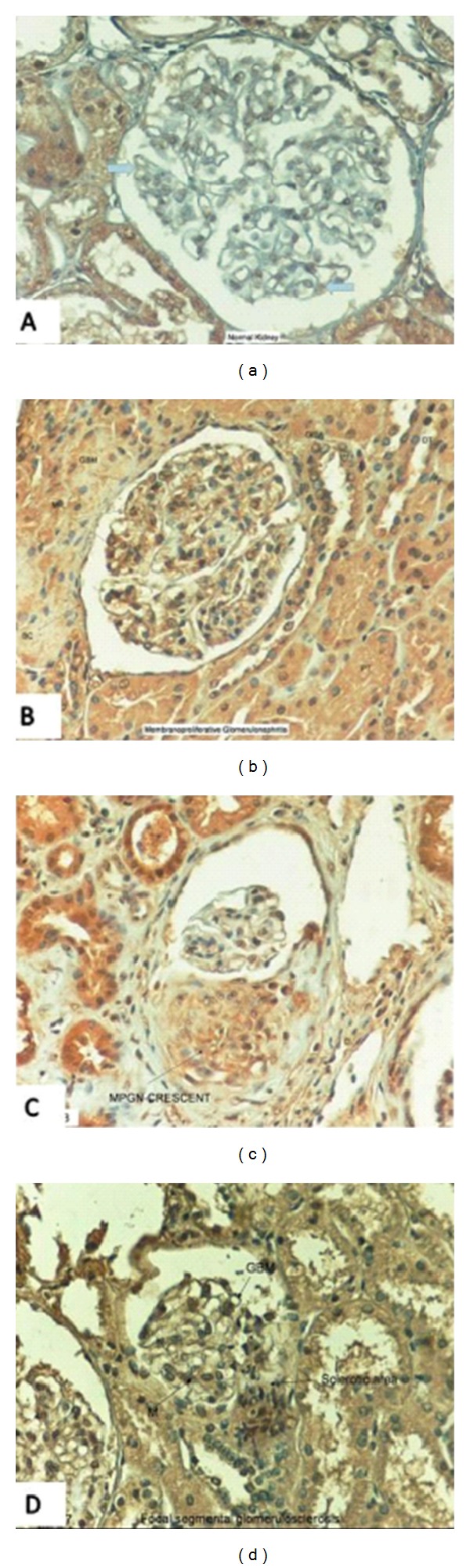
(a) Localization of urotensin-II (U-II) immunoreactivity (brown color) in the normal human kidney. Weak immunostaining in glomerulus, abundant expression of U-II in tubules. Thick arrows: weak immunostaining and black arrow: strong immunostaining [21]. (b) Urotensin-II immunoreactivity in membranoproliferative glomerulonephritis. Immunostaining in glomerular basement membrane, mesangium, Bowman capsule, and tubules. GBM: glomerular basement membrane, MPGN: membranoproliferative glomerulonephritis, BC: Bowman capsule, M: mesangium, PT: proximal tubule, and DT: distal tubule [21]. (c) Localization of urotensin-II immunoreactivity in a crescent [21]. (d) Urotensin-II immunoreactivity in focal segmental glomerulosclerosis. Notice the abundant expression of U-II in sclerotic area [32].

Shenouda et al. [20] demonstrated that U-II was mostly present in the epithelial cells of tubules and ducts, with greater intensity in the distal convoluted tubules in normal human kidneys. Moderate U-II immunoreactivity was seen in the endothelial cells of renal capillaries, but only focal immunoreactivity was found in the endothelial cells of the glomeruli. We also observed similar results in kidneys of children [21], and these findings suggest that hU-II may contribute to the pathophysiology of human kidneys (Figure 1(a)) [21, 32]. 

Expression of U-II mostly in tubules may suggest its probable active role in renal hemodynamics. Loretz and Bern [33] demonstrated that U-II stimulated Na transport in the teleost urinary bladder, while Ovcharenko et al.'s study [34] indicated that short-term adminisration of U-II did not influence sodium (Na) handling by the kidney in rats. However, Zhang et al. [35] observed that infusion of U-II directly into the rat renal artery increased renal blood flow (RBF), associated with a diuresis and natriuresis. In contrast, Song et al. [11] reported that U-II caused an antinatriuresis and antidiuresis when administered as an i.v. bolus dose and stressed that this was associated with and driven by renal hemodynamic effects leading to a marked reduction in glomerular filtration rate (GFR).

Two years later from the above study, Abdel-Razik et al. [36] searched the potential direct tubular action of urotensin in rats. They observed dose-dependent changes in GFR and urinary electrolytes. The hemodynamic effects were predominated at higher doses and caused a profound reduction in GFR which was accompanied by an antidiuresis and anti-natriuresis. When a lower infusion rate of rat U-II was employed, a tubular action to reduce electrolyte reabsorption became apparent through an increase in fractional excretion of Na and potassium (K) [36]. 

However, in children with minimal change nephrotic syndrome (MCNS), and their healthy controls, we could not find any relationship between the U-II level and Na/K excretion [24]. Although this differences may be partially related to different biological effects of U-II in different species, contradictory observations in same species underline the complex influence of U-II on renal hemodynamics. 

It is not clear enough whether the effect of U-II on tubular transport is direct or mediated by secondary mechanisms. However, considering the expression of U-II receptor in the thin ascending limb of Henle's loop and the inner medullary collecting duct [11] and the greater U-II receptor mRNA and protein expression in the medulla compared with the cortex [36], together with the abundant expression of hU-II in the proximal and distal tubules of children (patients and controls) [21], it may be suggested that U-II may have a direct action on tubular electrolyte transport. 

## 3. The Effect of Urotensin-II on Kidney Diseases 

Since U-II and its receptor, GPR14, are expressed abundantly in cardiorenal system [10, 11], most of the researches on it are related to cardiovascular and renal diseases. 

Although some studies have been investigated the circulating levels of U-II in several diseases, such as hypertension [37], congestive heart failure [38], renal failure [3], MCNS [24], and preeclampsia-eclampsia [39], little is known about the actions of this important peptide within the kidney. Some studies suggest that renal dysfunction affects the U-II levels, since the plasma U-II level has been found elevated in renal failure [3], congestive heart failure [38], and systemic hypertension [37], and it was found to be an inverse predictor of overall and cardiovascular mortality in patients with moderate-to-severe chronic kidney disease (CKD) [40]. 

Certain diseases, such as hypertension and glomerulonephritis, may alter the expression of U-II. It has been shown that both U-II and its receptor mRNA expression levels were up to threefold higher in spontaneously hypertensive rat (SHR) tissue compared to control Wistar-Kyoto (WKY) rats, taking into consideration that SHR is more sensitive than WKY to the effect of U-II [41]. 

In the literature, there are no enough data on the level of this vasoactive peptide in glomerular diseases. Recently, we firstly demonstrated that U-II was present in plasma and urine samples of 26 children with MCNS [24]. It showed important changes in relapse and remission periods. Plasma U-II concentrations during relapse were significantly lower than in remission and in controls, whereas urinary U-II levels were higher in relapse than in remission [24]. The plasma U-II level showed a significant positive correlation with the plasma albumin concentration during remission. However, there was no correlation between the amount of proteinuria and plasma/urinary U-II levels, and we could not detect any relationship between U-II levels and other clinical and laboratory parameters (such as the age at onset of disease, number of relapses, time to remission, blood pressure, serum creatinine, and hematological parameters). We suggested that the important changes in plasma and urinary U-II levels during relapse may be the result of heavy proteinuria rather than playing a role in mediating the clinical and laboratory manifestations of MCNS. After this, it would be interesting to search the possible role(s) of this peptide in children with glomerular diseases other than MCNS. Therefore, we examined the urotensin-II immunoreactivity in renal biopsy specimens of children with several renal diseases, including membranoproliferative glomerulonephritis (MPGN), membranous nephropathy (MGN), IgA nephropathy (IgAN), Henoch-Schönlein nephritis (HSN), and focal segmental glomerulosclerosis (FSGS) [21, 32]. In normal human kidney, there was weak expression of human U-II in glomerulus, while abundant expressions were seen in proximal, distal tubules, and collecting ducts (Figure 1(a)) [21], similar to a previous study [20]. 

We observed different expression pattern of U-II in different glomerular diseases. In MPGN and FSGS, different from the normal kidneys, more dense U-II immunoreactivity was seen in the glomerular basement membrane (GBM), glomerular mesangium, Bowman capsule (BC), and tubules (Figures 1(b), and 1(d)) [21, 32]. Interestingly, we also observed U-II immunoreactivity in crescents (Figure 1(c)), and sclerotic areas in FSGS (Figure 1(d)) [21, 32]. 

Systolic blood pressure (BP) was positively correlated with mesangial expression of U-II (*r* = 0.418,  *P* = 0.042), while diastolic BP was correlated with endothelial U-II expression in MPGN (*r* = 0.469,  *P* = 0.021) [21]. 

In children with MGN, U-II was mostly seen in GBM and BC. We observed more dense U-II immunoreactivity in distal tubules (*P* = 0.030), endothelium (*P* = 0.009), and mesangium (*P* = 0.002) in children with MPGN than in MGN. Diastolic BP was positively correlated with the expression of U-II in BC in children with MGN (*r* = 1,  *P* = 0.000) [21]. 

There is no enough data about the precise role of hU-II in renal diseases, and that was the first report demonstrating the presence of U-II by immunohistochemically in children with several renal diseases, suggesting that hU-II may contribute to the pathophysiology of human kidneys.

The positive correlation between BP and intensity of U-II expression in mesangium and endothelium in MPGN, and BC in MGN was noteworthy. Considering the literature data about U-II, as an important physiological mediator of vascular tone and blood pressure in humans [16], and also an extremely potent constrictor of renal blood vessels from primates [6], it is reasonable to suggest that U-II may play an important role in the regulation of BP in MPGN and MGN. 

As it has been known, mainly, two basic mechanisms are feasible in glomerulonephritis: antibody interaction with antigens in situ within the glomerulus and antibody binding to soluble antigens in the circulation, followed by immune-complex deposition within the glomeruli [42]. The secondary immune mechanisms of glomerular injury are the cascade of inflammatory mediators that are recruited to propagate renal damage following the primary glomerular attack. Some of these mediators play essential roles, whereas others may aggravate the glomerular lesion [42]. Most of the secondary mediators include cytokines, growth factors, reactive oxygen metabolites, bioactive lipids (platelet-activating factor and eicosanoids), proteases, and vasoactive substances, such as ET and NO [42]. 

Since U-II is abundantly expressed in the glomeruli in MPGN and MGN, it is reasonable to suggest that U-II may play a role in this mechanism, probably in the secondary immune mechanisms of glomerular injury, by a paracrine or an endocrine action [21]. Djordjevic et al. [43] demonstrated that hU-II increases the levels of NADPH oxidase-derived reactive oxygen species, leading to the activation of mitogen-activated protein kinases and protein kinase B (akt), followed by enhanced plasminogen activator inhibitor-1 expression and increased proliferation of pulmonary arterial smooth muscle cells. It has been also shown that exposure of the rat proximal tubular epithelial cells (NRK-52E) to transforming growth factor *β*1 (TGF-*β*1) or angiotensin II (Ang II) increased U-II and GPR14 mRNA expressions [44], and U-II acts synergistically with Ang II [45, 46]. As might be expected, oxidative stress, mediators, and inflammation are like a devil's triangle in kidney diseases, mostly they induce each other. Since there is a complex relationship between U-II and oxidative stress [43], and other mediators, such as TGF-*β*1 and Ang II [44–46], U-II is probably more than a mediator in glomerular diseases and takes place in an important part of this devil's triangle. 

Interestingly, we observed abundant U-II immunoreactivity in crescents and sclerotic areas in FSGS [21, 32]. Crescents are composed of large swollen cells arising from both macrophages of hematogenous origin and native parietal epithelial cells [47]. As time elapses, the cellular crescents are progressively replaced by fibroblasts, and in more advanced stages, the fibroblastic component is entirely replaced by collagenous lamellar materials with a few remnant cells [48]. Recent reports have shown a mitogenic role for U-II through induction of smooth muscle cell proliferation [49, 50], and additionally, it has been shown to induce collagen deposition by fibroblasts [51]. Zhang et al. [52] showed that U-II could stimulate the phenotypic conversion, migration, and collagen synthesis in adventitial fibroblasts. Additionally, it may act as autocrine/paracrine growth stimulators in tumor cells [53]. 

The pathogenesis of glomerulosclerosis is still unknown. Several factors, cytokines and growth factors, hyperlipidemia and platelet activation, lead to an increase of mesangial matrix production by resident cells. Several data demonstrate that abnormal glomerular growth is associated with glomerular sclerosis [54]. 

Since hU-II stimulates cell proliferation in adrenal tumors [55], renal epithelial cells [23], and vascular smooth muscle cells [49] and it has been found elevated in carotid and aortic atherosclerotic plaques [56], the abundant expression of U-II in crescents and sclerotic areas suggests that U-II may also play a role in the progression of crescents and glomerular sclerosis, probably as a growth factor or as an inflammatory peptide. This hypothesis must be searched and tested in future. 

MGN is an antibody-mediated disease of uncertain and imprecise pathogenesis. However, the hypotheses that it is an autoimmune disease of the kidney and that the subepithelial immune deposits are formed in situ with an endogenous glomerular antigen are attractive [57]. The electron-dense deposits are generally located at the site of the slit diaphragm, and subepithelial space, while no electron-dense deposits are seen in the subendothelial space or in the mesangium, and hypertension at onset is associated with a less favorable outcome in MGN [57]. In our study, hU-II expression was mostly seen in GBM and BC, and there was a strong positive correlation between diastolic BP and intensity of U-II expression in BC. These findings may increase two interesting questions: may U-II play a role in the formation of these deposits as a mitogenic factor, as we mentioned previously, and may it have any role in the clinical course of MGN by regulating the BP? However, it is difficult to answer these questions with that study, and these hypothesis must be clarified by further detailed studies. 

In kidneys of children with HSN and IgAN, similar to each other, more dense U-II immunoreactivity was seen in GBM, glomerular mesangium, BC, proximal/distal tubules, and also in crescents [21, 32]. 

Although the pathogenesis of IgAN and HSN is not well known [58], animal studies have shown the key role of cytokines and growth factors (particularly platelet-derived growth factor and TGF-*β*) in the induction and resolution of mesangial injury, and there is some evidence that these are also involved in IgAN [58]. The similar expression pattern of U-II in HSN and IgAN has been considered that U-II may have a role in mesangial inflammation and crescent formation in these disorders [32].

Different expression pattern of U-II in several renal diseases may give rise to thought whether the effect of U-II gene polymorphism. Recently, we performed a preliminary study, and firstly investigated the possible association between a coding single nucleotide polymorphism of UT-II gene, T21M (T/C), in 87 children with childhood nephrotic syndrome (NS), 16 children with acute poststreptococcal glomerulonephritis (APSGN), and 10 children with HSN [59]. We found higher TC genotype of U-II gene in NS (56.3% versus 38.9%, *P* = 0.025), higher TT genotype in APSGN (50.0% versus 25.9%, *P* < 0.001), and a positive correlation between TT polymorphism and the presence of macroscopic hematuria in APSGN (*r* = 0.51, *P* = 0.04). This study considered that urotensin-II may be an important mediator in pathophysiology of the childhood glomerulonephritis, and Turkish children with TC genotype may have a higher genetic susceptibility to NS, while TT genotype of U-II may increase the risk of APSGN. 

## 4. Urotensin-II Antagonists as a New Promising Pharmacological Treatment Target

Several influences of U-II in cardiovascular/renal system, and the presence of its receptor in the heart, lungs, blood vessels, kidneys, and brain, led the researchers to investigate the role of U-II antagonists in various diseases. The most known U-II receptor antagonists are palosuran and urantide. Sidharta et al. [60] investigated whether palosuran, a potent, selective, and competitive antagonist of the U-II receptor, had effects in macroalbuminuric, diabetic patients who are prone to the development of renal disease. They observed an overall clinically significant reduction of 24.3% in the 24-hour urinary albumin excretion rate. 

In an experimental study, it has been shown that long-term treatment of streptozotocin-induced diabetic rats with palosuran improved survival, increased insulin, and slowed the increase in glycemia, glycosylated hemoglobin, and serum lipids. Furthermore, palosuran increased renal blood flow and delayed the development of proteinuria and renal damage [61]. 

These two researches suggest that U-II receptor antagonism might be a new therapeutic approach for the treatment and/or prevention of diabetic nephropathy.

The tolerability and safety, pharmacokinetics, and pharmacodynamics of palosuran were evaluated in also healthy young men with a double-blinded placebo-controlled single ascending dose designed study [62]. It has been shown that palosuran was well tolerated, and no serious adverse events or dose-related adverse events were reported. However, as the authors stressed, the results of this entry-into-humans study warrant further investigation of the therapeutic potential of palosuran.

Recently, Nitescu et al. [63] examined the effects of another selective U-II receptor antagonist, urantide, on renal hemodynamics, oxygenation, and function in endotoxemic rats. However, they found that urantide had no statistically significant effects on any of the investigated variables (kidney function, renal blood flow, cortical and outer medullary perfusion, and oxygen tension) in these rats.

 In spite of different results about the effectiveness of U-II antagonism, it appears that the therapeutic potential of U-II antagonists may be the focus of research interest in the near future.

In summary, U-II may be an important mediator, in fact probably more than a mediator, in kidney diseases. Whether the observed findings which are primary or secondary to these pathological conditions still remain unclear, they suggest a possible role of U-II in the pathophysiology of several kidney diseases. It looks like that U-II will continue to give new messages as well as raising more questions as research on it increases. Further, detailed studies are needed to address the exact role(s) of this peptide in renal diseases.
